# Delayed Presentation of Thoracic Aortic Pseudoaneurysm Following Pedicle Screw Implantation: A Case Report

**DOI:** 10.1111/os.12793

**Published:** 2021-01-06

**Authors:** Li‐di Liu, Xin Hong, Jiang‐bi Li, Shao‐kun Zhang

**Affiliations:** ^1^ Department of Spine Surgery The First Hospital of Jilin University Changchun China; ^2^ Department of Vascular Surgery Japan Union Hospital of Jilin University Changchun China

**Keywords:** Aortic injury, Malposition, Pedicle screw implants, Pseudoaneurysm formation, Thoracic and lumbar spine

## Abstract

**Background:**

Pedicle screw insertion has been known to have several complications even in the most skilled surgical hands. However, injury to the thoracic aorta during pedicle screw insertion is rare, delayed presentation secondary to pseudoaneurysm is even rarer, the pseudoaneurysm formation caused by a series of malpositioned pedicle screws has perhaps not been reported so far.

**Case presentation:**

In this paper, we report here a case in which inadvertent injury to the thoracic aorta resulted in pseudoaneurysm, its manifestation was initially vague, resulting in a delayed diagnosis. Delayed aortic pseudoaneurysm or injury can be asymptomatic for a long time. Patients with renewed or continued back pain should alert orthopaedic surgeons regarding the possibility of pseudoaneurysms, regardless of the period that has elapsed after pedicle screw implantation.

## Graphical abstract

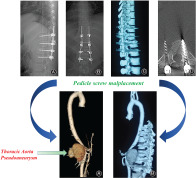

## Introduction

Pedicle screw insertion is one of the most commonly performed spinal surgical procedures. Given the fact that the instrumentation results in stabilization of all three spinal columns, its use is considered the gold standard while performing fixation for various spinal conditions. Iatrogenic pseudoaneurysm is a rare and dangerous complication during pedicle screw instrumentation surgeries^1^, ^2^. Major vascular injuries of lumbar disc surgeries have an incidence of less than 0.01%. In rare cases, inadvertent errors could lead to screws impinging on the aorta, leading to a risk of perforation or pseudoaneurysm formation due to the constant pulsation of the aorta against the hardware^3^, ^4^.

We present a case of aortic pseudoaneurysm formation after pedicle screw instrumentation and reviewed the available literature about such injuries. Key learning points, management techniques, and avoidance are discussed.

## Case Report

A 21‐year‐old man presented to the emergency room (ER) complaining of stomach and serious back pain 2 months after spinal surgical intervention. Two months before his visit, he fell from a 10 m tall building. He was confirmed as suffering fracture and dislocation from T_11_ to L_2_ by a computed tomography (CT) examination. The patient complained of severe back pain. There was no neural deficit. The patient underwent thoracolumbar pedicle screw implant surgery using ‘freehand technique’ under fluoroscopic guidance in a local hospital. Eight pedicle screws with contoured rods were placed spanning T_11_ to L_2_ after the correction of deformities. The patient tolerated the procedure well and developed no neurodeficits postoperatively. Radiological examination revealed a partial correction of the deformity.

The patient was presented with a recurrence of pain at the operated site 10 days postoperatively. The pain was a dull ache, non‐radiating, and showed a progressive increase in intensity. This was accompanied by constitutional symptoms like poor appetite and a general feeling of being unwell. In view of the non‐specific nature of the complaints, no further investigation was carried out and the patient was advised to attend regular follow‐ups.

**Fig. 1:**
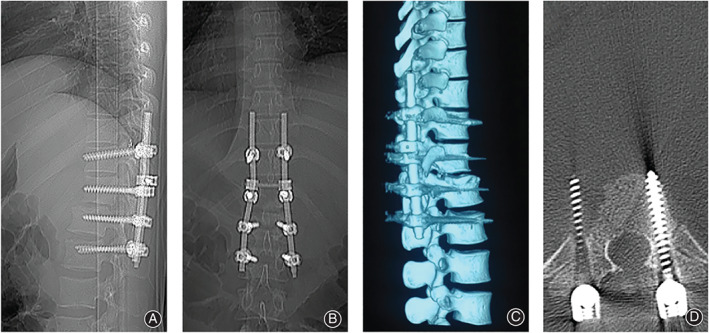
Plain radiographs after pedicle screw implant surgery. The dislocation of T_12_‐L_1_ was partly corrected (A). Both sides of the T_11_ and T_12_ pedicle screws seemed to have less TSA than the ones in the L_1_ and L_2_ vertebrae (B). Three‐dimensional reconstruction CT also showed the malplacement on the right side of the pedicle screw in L_2_ vertebra (C).Thoracolumbar CT shows malplacement of pedicle screws at T_12_ levels, which penetrated the aortic wall and formed a pseudoaneurysm of the aorta (D).

**Fig. 2:**
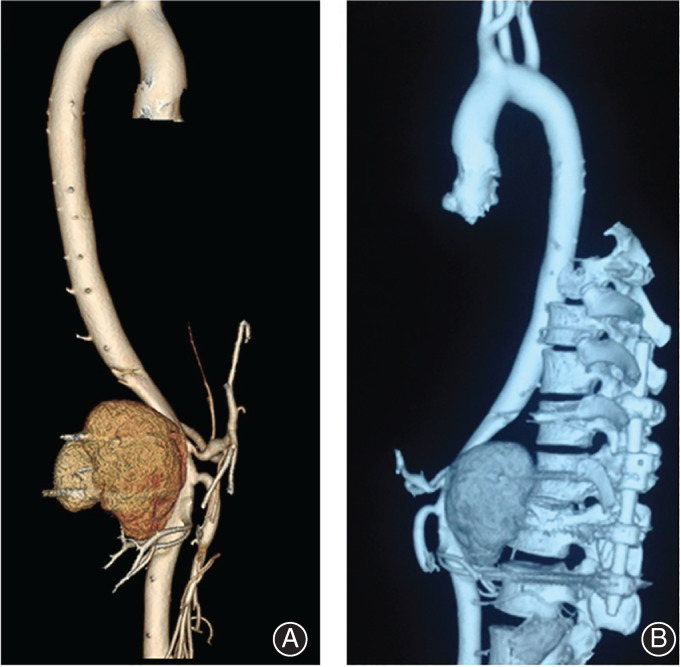
Revascularization of the aortic 2 months after pedicle screw implant surgery, which showed the aortic pseudoaneurysm formation (A). Contact of the pedicle screws and the pseudoaneurysm (B).

In our hospital, enhanced CT and X‐rays were performed after thorough clinical evaluation. The CT scan revealed the malposition of a pedicle screw on both sides at the T_11_, T_12_, and L_1_ levels, which was a disaster. The left side T_12_ and L_1_ malpositioned screws had exited the lateral pedicle cortex and were clearly abutting the posteromedial aspect of the descending thoracic aorta (Fig. 1), with the formation of associated pseudoaneurysms in the thoracic aorta. The right side of T_11_ and both sides of T_12_ screws also had exited the lateral pedicle cortex and were abutting the wall of the pseudoaneurysm. The left side screw in L_2_ perforated the lateral pedicle cortex but did not abut the aorta (Fig. 2). A re‐surgery with the interdisciplinary collaboration of orthopaedics and vascular surgery teams was recommended and was subsequently performed in another hospital. In the surgery, after the anesthesia and cardiopulmonary bypass, pseudoaneurysm and malpositioned screws were found under direct vision. An ascending aorta replacement with artificial tissue was performed (Vascutek Ltd., diameter 16 × 8 mm, usable length 45 cm). The extruded part of the pedicle screw was cut *in situ* so as to avoid any chance of re‐injury. The patient endured the surgery well and had a good recovery in his 4‐month follow‐up examination.

## Discussion

Acute aortic injuries caused by pedicle screw implantation have been reported previously^5^, ^6^. Delayed aortic injuries, which have been seldom reported, refer to those in which patients experience symptoms over 30 days postoperatively.

Delayed aortic injuries have a different clinical presentation from acute ones (Table 1). In acute cases, the presentation is quite dramatic with a sudden intraoperative drop in pressures and signs of acute hypovolemia that needs immediate resuscitation with fluids and control of bleeding. On the other hand, the delayed diagnosis of aortic injuries ranges from 30 days to 20 years^7^. Renewed back pain, either continued or serious, can be the first sign of this rare complication^6^, ^8^, ^9^. Special symptoms also include intermittent, pulsating epigastric pain, and asymmetric lower extremity arterial pulses. Spinal surgeons should exercise a degree of caution after the pedicle screw placement in the event of patients presenting any or all of the symptoms even after significant time has elapsed following the initial surgery. To our knowledge, this is the first case report about pseudoaneurysm formation caused by a series of malpositioned pedicle screws.

**TABLE 1:** Summary of studies on delayed aorta injuries after pedicle screw implants

Reference	Age/sex	Indication	Clinical presentation	Diagnosis delay (month)	Screw implant levels	Screw malplacement levels	Left or right
Choi *et al*.^1^, 2001	50/M	Kyphoscoliosis	Severe stinging back pain	14	NK	T_6_	Left
Wegener *et al*.^9^, 2008	69/F	T_9_ metastasis	Renewed back pain, intermittent and pulsating epigastric pain	12	T_7,8,10,11_	T_11_	Left
Kakkos *et al*.^2^, 2008	51/M	Tuberculous spondylodiscitis	Incidental finding	13	T_11,12_,L_2_	T_11_	Left
Watanabe *et al*.^7^, 2010	57/F	Osteoporotic T_12_ vertebral fracture	Postoperative routine finding	1	T_10_‐L_2_	T_10_	Left
Lopera *et al*.^6^, 2010	60/F*	Thoracic fracture	Chest pain, hemothorax hypotension	12	NK	NK	NK
	55/M	Thoracic fracture	Persistent back pain	3	NK	NK	NK
	52/M	Thoracic fracture	Incidental finding	3	NK	T_11_	NK
Tschoeke *et al*.^8^, 2011	64/F	Multiple myeloma	Progressive back pain	24	T_5,6,8,9_	T_5_,T_6_	Left
Freyrie *et al*.^17^, 2013	55/M	T_6,7_ fracture	Thoracic back pain Lower extremity arterial pulses	6	T_3,4,5,8,9_	T_4,5_	Left
Our cases, 2014	21/M	T_12_‐L_1_ fracture and dislocation	Gradually increased back pain	2	T_11_‐L_2_	T_11_,T_12_	Left

Diagnosis delay, the time between diagnosis of aortic injury and pedicle screw implants surgery; NK, not known

* Died on the way to OR.

The aorta extends along left side of the thoracic and lumbar vertebrae. Quite expectedly, in this case and other studies (Table 1), aortic injury happened with malpositioned left pedicle screws only. In previously reported cases as well as ours, the primary cause of displacement is the small transverse screw angle (TSA). A smaller TSA will make the pedicle screw prone to injuring the aorta. The second cause is that the surgeons did not confirm the integrity of the pedicle screw channels before implantation. The integrity of the anterior wall of the channels is especially critical for the prevention of aortic injuries.

Delayed aortic injuries could present over a wide spectrum and include perforation, pseudoaneurysm formation, and even complete ruptures of arterial walls, which could be fatal^6^, ^10^. In an *in vivo* bovine study, Faro *et al*. evaluated histological and biomechanical changes in thoracic aorta wall tissue resulting from intentional severe vessel impingement. During the 12‐month follow‐up period, the authors demonstrated that marked vessel impingement resulted in acute and chronic histopathological changes that significantly compromised vessel wall integrity on biomechanical testing^11^.

For malpositioned screws with obvious injured aortic walls, revision surgery is needed. But the potential risk of complete rupture of the aorta should be considered and appropriate strategizing is required. Choi *et al*. reported a case with malpositioned pedicle screws 14 months after surgery. The surgical team re‐intervened and removed the malpositioned screw from the T_6_ vertebra. Three weeks later, the patient suffered fever and hemorrhagic shock and was diagnosed as having a pseudoaneurysm and bleeding from the aorta. Subsequently, the patient had to undergo re‐exploration anteriorly with vascular surgery^1^. These and our cases suggest that a preoperative interdisciplinary evaluation is recommended, even under emergency conditions^12^.

Controversy exists in the accidental finding of asymptomatic patients. Sometimes, screws have slight contact with the aorta. The integrity of the aortic wall may not have been compromised in such cases. When such screws are seen in postoperative imaging in an asymptomatic patient, the surgeon must decide whether it is riskier to revise the screw or to not do any intervention and merely observe it.

Foxx retrospectively reviewed 182 consecutive patients, 680 pedicle screws undergoing spinal implants, and found 33 screws were in contact with a major vessel on routine postoperative imaging. Foxx had a mean follow‐up of 44 months and revealed no detectable vessel abnormality in subsequent follow‐ups. He recommends consideration of the overall life expectancy of the patient before contemplating any intervention^13^.

Some surgeons removed only the screws that show higher potential risk^14^, ^15^. Parker *et al*. suggested that it is not mandatory to remove all pedicle screws found to be encroaching on major vascular structures. In cases in which the screw is not revised, regular radiographical follow‐up is required to detect the formation of a delayed pseudoaneurysm or another secondary vascular injury^16^. With a similar concept and idea, Freyrie *et al*. followed up a patient with malpositioned pedicle screws for 6 months. Unfortunately, the screw penetrated the thoracic aortic wall, and a vascular surgery had to be performed^17^. Thus, considering the risk of aortic rupture, a multidisciplinary surgery strategy should be prepared before the removal of pedicle screws abutting the aorta for asymptomatic patients.

## Conclusion

Delayed aortic pseudoaneurysm or injury can be asymptomatic for a long time. Patients with renewed or continued back pain should alert orthopedic surgeons regarding the possibility of pseudoaneurysms, regardless of the period that has elapsed after pedicle screw implantation.
